# Novel Architecture Titanium Carbide (Ti_3_C_2_T_x_) MXene Cocatalysts toward Photocatalytic Hydrogen Production: A Mini-Review

**DOI:** 10.3390/nano10040602

**Published:** 2020-03-25

**Authors:** Van-Huy Nguyen, Ba-Son Nguyen, Chechia Hu, Chinh Chien Nguyen, Dang Le Tri Nguyen, Minh Tuan Nguyen Dinh, Dai-Viet N. Vo, Quang Thang Trinh, Mohammadreza Shokouhimehr, Amirhossein Hasani, Soo Young Kim, Quyet Van Le

**Affiliations:** 1 Department for Management of Science and Technology Development, Ton Duc Thang University, Ho Chi Minh City 700000, Vietnam; 2 Faculty of Applied Sciences, Ton Duc Thang University, Ho Chi Minh City 700000, Vietnam; 3 Key Laboratory of Advanced Materials for Energy and Environmental Applications, Lac Hong University, Bien Hoa 810000, Vietnam; nbsonhd@gmail.com; 4 Department of Chemical Engineering, R&D center for Membrane Technology and Research Center for Circular Economy, Chung Yuan Christian University, Chungli Dist., Taoyuan City 32023, Taiwan; chechiahu@cycu.edu.tw; 5 Institute of Research and Development, Duy Tan University, Danang 550000, Vietnam; nguyenchinhchien@duytan.edu.vn (C.C.N.); dltnguyen@yahoo.com (D.L.T.N.); 6 Faculty of Environmental and Chemical Engineering, Duy Tan University, Da Nang 550000, Vietnam; 7 Faculty of Chemical Engineering, University of Science and Technology, The University of Da Nang, 54 Nguyen Luong Bang, Da Nang 550000, Vietnam; ndmtuan@dut.udn.vn; 8 Center of Excellence for Green Energy and Environmental Nanomaterials (CE@GrEEN), Nguyen Tat Thanh University, 300A Nguyen Tat Thanh, District 4, Ho Chi Minh City 755414, Vietnam; daivietvnn@yahoo.com; 9 Cambridge Centre for Advanced Research and Education in Singapore (CARES), Campus for Research Excellence and Technological Enterprise (CREATE), 1 Create Way, Singapore 138602, Singapore; qttrinh@ntu.edu.sg; 10 Department of Materials Science and Engineering, Research Institute of Advanced Materials, Seoul National University, Seoul 08826, Korea; mrsh2@snu.ac.kr; 11 School of Chemical Engineering and Materials Science, Chung-Ang University, Seoul 06974, Korea; amirhossein.hasani88@gmail.com; 12 Department of Materials Science and Engineering, Korea University, 145 Anam-ro, Seongbuk-gu, Seoul 02841, Korea

**Keywords:** photocatalysis, Ti_3_C_2_T_x_, MXenes, photocatalysis, water splitting, HER

## Abstract

Low dimensional transition metal carbide and nitride (MXenes) have been emerging as frontier materials for energy storage and conversion. Ti_3_C_2_T_x_ was the first MXenes that discovered and soon become the most widely investigated among the MXenes family. Interestingly, Ti_3_C_2_T_x_ exhibits ultrahigh catalytic activity towards the hydrogen evolution reaction. In addition, Ti_3_C_2_T_x_ is electronically conductive, and its optical bandgap is tunable in the visible region, making it become one of the most promising candidates for the photocatalytic hydrogen evolution reaction (HER). In this review, we provide comprehensive strategies for the utilization of Ti_3_C_2_T_x_ as a catalyst for improving solar-driven HER, including surface functional groups engineering, structural modification, and cocatalyst coupling. In addition, the reaming obstacle for using these materials in a practical system is evaluated. Finally, the direction for the future development of these materials featuring high photocatalytic activity toward HER is discussed.

## 1. Introduction

To date, sustainable solar hydrogen (H_2_) production, which directly produces by utilizing semiconductor photocatalysts, could provide a promising and environmental-friendly approach to solve the worldwide energy issues and reduce the dependence on fossil fuels [1,2]. Particularly, enormous progress has been made in developing a new system of photocatalysts such as transition metal dichalcogenides [3,4,5,6,7,8,9,10,11,12,13,14,15], transition metal oxide (TMOs) [16,17], transient metal sulfides (TMSs), graphitic carbon nitride (*g*–C_3_N_4_) [18,19,20,21,22], metal–organic framework (MOFs) [23,24,25], transition metal nitride (TMNs) [26], and transition metal carbide (TMCs) [27,28,29,30] that could efficiently enhance the H_2_ production, and readily scale up for commercialization [2]. 

**Figure 1:**
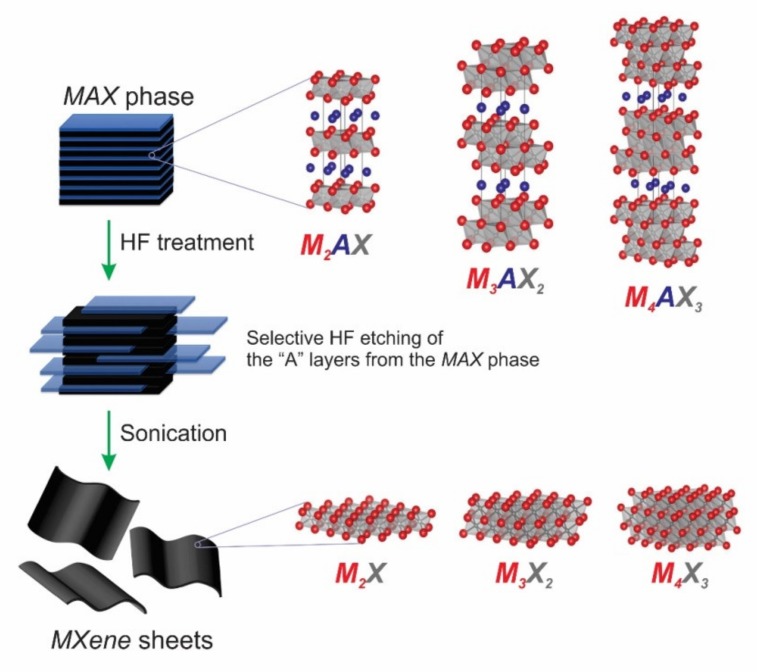
The schematic diagram is representing the process of synthesizing MXenes from MAX phases. Reproduced with permission from [31]. Copyright Wiley-VCH, 2014 and [32] Copyright American Chemical Society, 2012.

As an advanced and broad group of novel nanostructured materials, MXenes has been discovered and synthesized from the parent layered solids MAX phases (as shown in Figure 1) [31]. 

**Figure 2:**
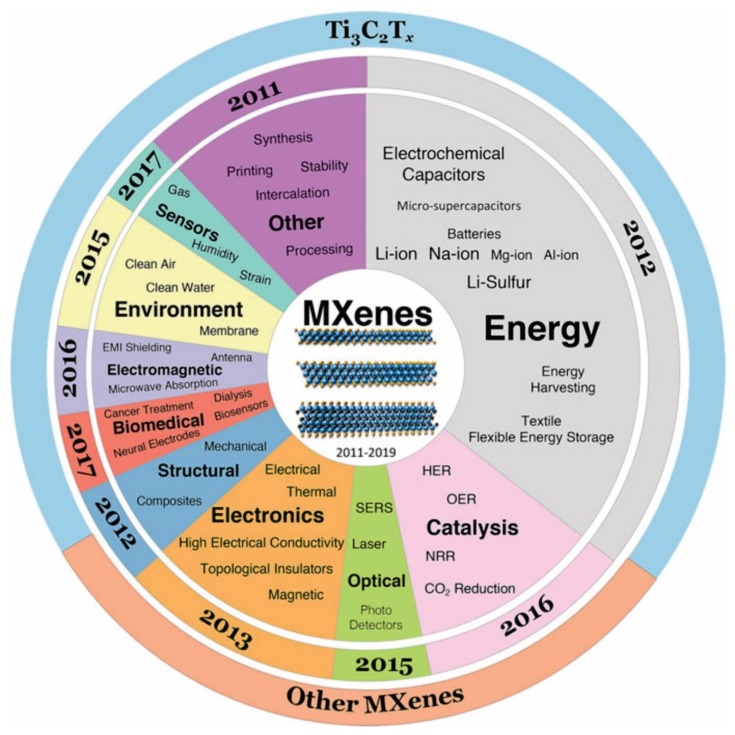
The general applications and properties of MXenes. The center pie chart explored the applications and properties of MXenes. The starting year in the middle pie chart ring indicates the exploration time of each application/property. The outer ring shows the ratio of publications, which were taken from 2011 to 2019 on the Web of Science, with the term of Ti_3_C_2_T_x_ versus the publications deal with all MXene compositions (M_2_XT_x_, M_3_X_2_T_x_, and M_4_X_3_T_x_). Reproduced with permission from [40]. Copyright Springer Nature, 2019.

**Figure 3:**
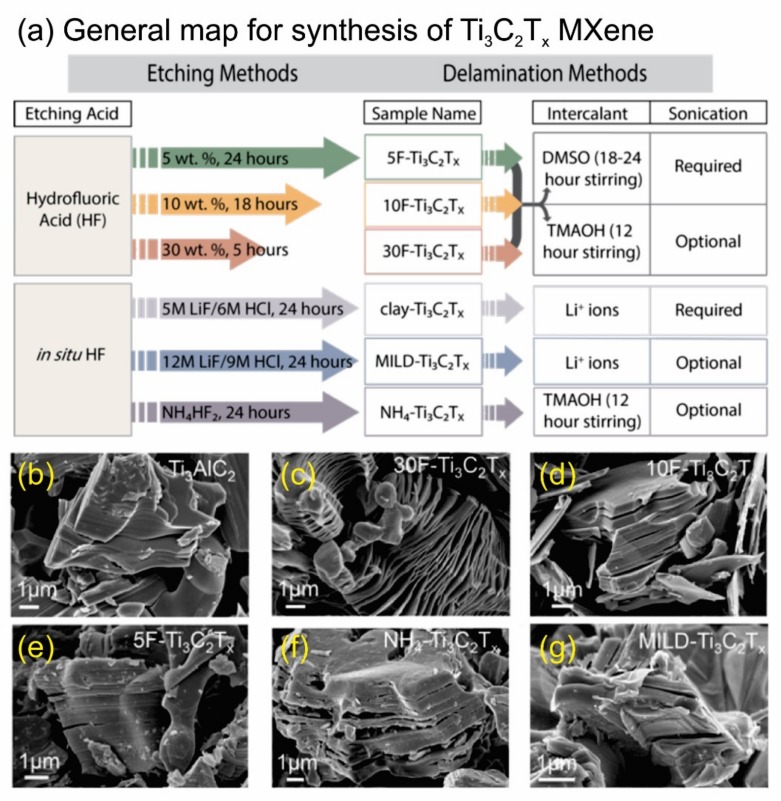
(**a**) The schematic diagram representing the process to prepare Ti_3_C_2_T_x_ by using different etchants (HF and in situ HF) and delamination methods and (**b**–**g**) their corresponded scanning electron microscopy (SEM) images. Reproduced with permission from [37]. Copyright Royal Society of Chemistry, 2019.

In essence, the chemical formula of MAX phases is M_*n*+1_AX*_n_*, which is defined by Barsoum [33,34,35,36,37]. In detail, the M element stands for transition metals from groups 3 (Sc), 4 (Ti, Zr, and Hf), 5 (V, Nb, and Ta), and 6 (Cr and Mo), the A element represents from groups 12 (Cd), 13 (Al, Ga, In, and Tl), 14 (Si, Ge, Sn, and Pb), 15 (P and As), or 16 (S), and the X element is C and/or N [33,38,39]. MXenes are generally prepared by selectively getting rid of the element of A from the parent MAX phase to form M_*n*+1_X*_n_*T_x_ (*n* = 1–3), where T_x_ is the surface termination groups ((–O), (–F), and (–OH)) [31]. MXenes materials, which offer many advantages electronic, optical, plasmonic, and thermoelectric properties [36], have attracted much interest recently. They are currently explored for a variety of applications, including energy, environment, catalysis, photocatalysis, optical devices, electronics, biomedicals, sensors, electromagnetic, others, etc. (Figure 2) [40,41,42,43]. Among MXenes, many efforts have been devoted to promoting titanium carbide (Ti_3_C_2_T_x_) as the most promising candidate of cocatalysts [44,45,46]. Based on the published literature dealing with MXenes, which was taken from 2011–2019 on the Web of Science, there was about 70% of researches on MXenes associated with Ti_3_C_2_T_x_, as seen in the third ring of the pie chart in Figure 2 [40]. It also notes that the Ti_3_C_2_T_x_ also shows a high potential to replace the expensive Pt cocatalyst in photocatalysis. In 2017, Alhabeb et al. have provided an excellent report to give step-by-step guidance to preparing of Ti_3_C_2_T_x_ by using different etchants (HF and in situ HF) and delamination methods (Figure 3a) [38]. Their corresponded scanning electron microscopy (SEM) images were obtained and shown in Figure 3b–g. For detail, Ti_3_AlC_2_ sample show compactly layered morphology (Figure 3b), while the morphology of the multilayered Ti_3_C_2_T_x_ samples was influenced by weight percent (wt %) of HF (Figure 3c–e). On the other hand, the morphology of the multilayered NH_4_–Ti_3_C_2_T_x_ sample (Figure 3f) and MILD-Ti_3_C_2_T_x_ sample (Figure 3g) are structurally similar to that of 5F–Ti_3_C_2_T_x_ multilayered powders (Figure 3e). Theoretically, the Ti_3_C_2_Tx fulfill the prerequisite requirement condition for applications as catalysts for HER. It has been reported that the O and F terminated Ti_3_C_2_ are metallic based semiconductors with a conductivity up to 9880 S·cm^−1^, which is higher than that of graphene [47]. This indicates that the charge transfer between Ti_3_C_2_ to the active site is superior to most of the reported semiconducting catalysts. Furthermore, the H* adsorption energy on the surface of Ti_3_C_2_ is close to 0, making it the best among noble metal free catalysts for application in HER [48]. However, most MXenes including Ti_3_C_2_T_x_ are semiconductors with indirect bandgaps [49]. To apply as photocatalysts, T_3_C_2_T_x_ needs to pair with other photoactive materials such as TiO_2_, CdS, *g*–C_3_N_4_ and metal organic frameworks (MOFs). Although, the development of MXenes for wide-range application in recent years have been thoroughly summarized and discussed [34,35,36,49,50,51], a review that focuses on Ti_3_C_2_T_x_ for photocatalytic HER has not been reported yet.

In this review, we present the use of Ti_3_C_2_T_x_ as the most potential and promising cocatalysts toward photocatalytic hydrogen production. Based on the recent research works, the influence on different morphology (nanotubes, nanoscrolls, quantum dots, etc.), surface termination groups (–F, –OH, and –O), and photocatalyst systems (titania (TiO_2_), graphitic carbon nitride (*g*–C_3_N_4_) coupled Ti_3_C_2_ photocatalysts, etc.) are reviewed and intensified. Additionally, attention and outlook on critical challenges, prospects, and potential applications for Ti_3_C_2_T_x_ cocatalysts toward sustainable solar hydrogen production are also highlighted.

## 2. Coupled Morphological and Structural Ti_3_C_2_T_x_ Cocatalysts

Since the morphology of photocatalysts could directly influence the photocatalytic process, active sites, and charge transfer, various nanostructures of Ti_3_C_2_T_x_ photocatalysts have been explored to improve the efficiency of H_2_ production. However, it has not been shown yet, which types of morphology and structure of Ti_3_C_2_T_x_ cocatalysts perform the best photocatalytic H_2_ production rate. In this section, the photocatalytic activity over different morphological and structural Ti_3_C_2_T_x_ cocatalysts was adequately highlighted and critically evaluated in terms of the H_2_ production rate (μmol·g_cat_^−1^·h^−1^) for convenient comparative purposes.

**Figure 4:**
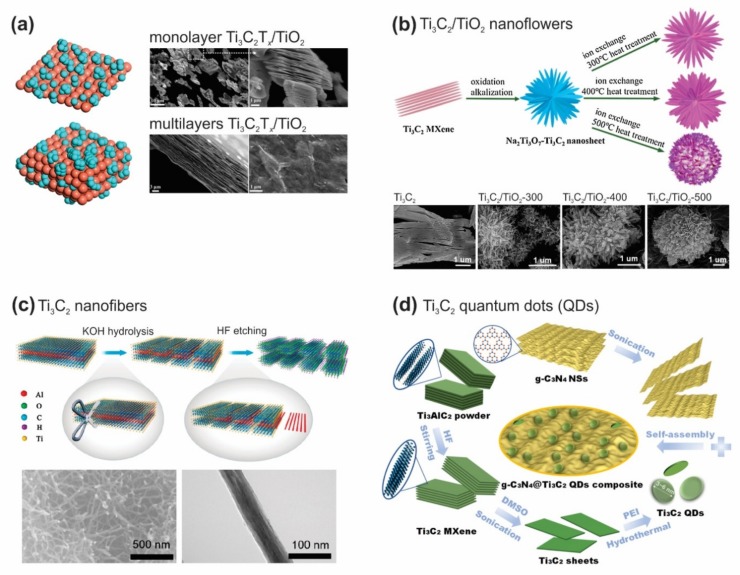
(**a**) Monolayer and multilayers Ti_3_C_2_T_x_ as the cocatalysts. Reproduced with permission from reference [52]. Copyright American Chemical Society, 2019; (**b**) the preparation of Ti_3_C_2_T_x_/TiO_2_ nanoflowers and their corresponding SEM images. Reproduced with permission from ref. [53]. Copyright Nature Publishing Group, 2018; (**c**) the preparation of Ti_3_C_2_T_x_ nanofibers and their corresponding SEM, TEM images. Reproduced with permission from ref. [54]. Copyright American Chemical Society, 2018; and (**d**) Schematic diagram for preparing of g–C_3_N_4_@Ti_3_C_2_T_x_ quantum dots composites. Reproduced with permission from ref. [55]. Copyright American Chemical Society, 2019.

Su et al. prepared a series of Ti_3_C_2_T_x_/TiO_2_ composite photocatalysts with a monolayer and multilayers Ti_3_C_2_T_x_ as the cocatalyst (as shown in Figure 4a) [52]. The result showed that a monolayer Ti_3_C_2_T_x_/TiO_2_ composite exhibited the superior H_2_ production rate (2650 μmol·g_cat_^−1^·h^−1^) under a 200 W Hg lamp integrated with a cutoff filter of 285–325 nm, which had more than nine-fold and two-fold higher, compared to the pure TiO_2_ (290 μmol·g_cat_^−1^·h^−1^) and multilayer counterpart (920 μmol·g_cat_^−1^·h^−1^), respectively. The enhancement of performance is possible due to the advanced electrical conductivity of a monolayer Ti_3_C_2_T_x_ and the effective charge-carrier separation at the Ti_3_C_2_T_x_/TiO_2_ interface. To propose a new morphology, Li et al. designed Ti_3_C_2_T_x_/TiO_2_ nanoflowers, which performed an outstanding H_2_ production rate, compared with that of pure TiO_2_ (as shown in Figure 4b) [53]. In detail, the Ti_3_C_2_T_x_/TiO_2_ nanoflower could reach to 526 μmol·g_cat_^−1^·h^−1^ in the H_2_ production rate under a 300 W Xe arc lamp, which was more than four-fold higher than that of the TiO_2_ nanobelts (121.82 μmol·g_cat_^−1^·h^−1^). It notes that under the same experimental conditions, the H_2_ production rate was 371.17 μmol·g_cat_^−1^·h^−1^ over the Pt/TiO_2_ nanosheet. It suggests that the noble metal-free Ti_3_C_2_T_x_ was considered as an alternative cocatalyst to replace the expensive and precious noble metals, such as Pt, Au, etc. To further boost the H_2_ production activity, Yuan et al. prepared the Ti_3_C_2_T_x_ nanofibers (NFs) structure by hydrolyzation and selective etching of Ti_3_AlC_2_ MAX ceramics (Figure 4c) [54]. Compared with traditional Ti_3_C_2_ flakes, the Ti_3_C_2_ NFs could provide a much higher BET (Brunauer–Emmett–Teller) surface area and expose more catalytic active sites, leading to enhanced H_2_ production activity, high cycling stability, and long-term viability. Very recently, Li et al. had successfully designed Ti_3_C_2_T_x_ quantum dots (QDs) by a self-assembly method, which their schematic synthesis of *g*–C_3_N_4_@Ti_3_C_2_T_x_ QDs composites was shown in Figure 4d [55]. As expected, *g*–C_3_N_4_@Ti_3_C_2_T_x_ QDs composites performed the best photocatalytic activity (5111.8 μmol·g_cat_^−1^·h^−1^) under artificial sunlight (300 W Xe arc lamp integrated with an AM-1.5 filter), which was nearly ten-fold higher than that of *g*–C_3_N_4_/Ti_3_C_2_T_x_ sheets (524.3 μmol·g_cat_^−1^·h^−1^). Compared to the traditional Ti_3_C_2_T_x_ sheets, Ti_3_C_2_T_x_ QDs offered more abundant active edge sites, and excellent electronic conductivity. Additionally, the photoexcited carriers in *g*–C_3_N_4_@Ti_3_C_2_T_x_ QDs composites could be effectively separated to rapidly take part in photocatalytic H_2_ production activity, leading to enhanced photocatalytic performance efficiently. Therefore, owing to excellent physical properties, *g*–C_3_N_4_@Ti_3_C_2_T_x_ QDs composites performed a remarkable enhancement in the photocatalytic H_2_ production rate of 5111.8 μmol·g_cat_^−1^·h^−1^, indicating its high potential to scale up and accelerate the H_2_ production via the green photocatalysis approach.

The synthesis of different morphologies of Ti_3_C_2_T_x_ cocatalysts was successfully proposed. Based on the recent studies, morphologies of Ti_3_C_2_T_x_ (nanotubes, nanoscrolls, quantum dots, etc.), which might provide more BET surface area to enrich the active adsorption sites, and inhibit the recombination of e^−^–h^+^ pairs, resulting in effective influence to the photocatalytic activity, high cycling stability, and long-term viability.

## 3. Modified Ti_3_C_2_T_x_ Cocatalysts with Surface Termination Groups 

In general, surface termination groups (–F, –OH, and –O) of Ti_3_C_2_T_x_, which are predominantly dependent on the synthesis methods, have profoundly altered their physicochemical properties [56]. Based on theoretical calculations, many studies suggested that surface termination groups strongly influence the stability, electronic, optical, and transport properties of Ti_3_C_2_T_x_ [57,58,59,60]. Due to improving the photocatalytic activity toward sustainable solar hydrogen production, there has been motivation to enhance and control the physicochemical properties of Ti_3_C_2_T_x_ through surface termination groups. Li et al. found that the Ti_3_C_2_T_x_/TiO_2_ hybrids, which synthesized through simple calcination of F-Ti_3_C_2_T_x_, exhibited potential photocatalytic activity. Its performance was two-fold higher than that of the Ti_3_C_2_T_x_/TiO_2_ hybrids with calcining OH-Ti_3_C_2_T_x_ [37]. On the other hand, Ran et al. used density functional theory (DFT) calculations for designing and exploring the potential of novel Ti_3_C_2_T_x_ nanoparticles as a promising H_2_ production cocatalyst [61]. They replaced the (–F) terminations by (–O)/(–OH) terminations by the hydrothermal treatment, and found that (–O)/(–OH) terminations play a notable role for photocatalytic activity. This result was consistent with the previous finding by Sun et al. [56], who observed significant enhancement of H_2_ production (88 μmol·g_cat_^−1^·h^−1^) over O-Ti_3_C_2_T_x_, compared to control samples. To further modify the surface termination groups of Ti_3_C_2_T_x_, Yang et al. successfully prepared O-Ti_3_C_2_T_x_/CdS hybrids through the radiofrequency oxygen plasma method (O_2_/N_2_, 2.2 Pa, 500 °C, 1400 W, 2.45 GHz, and 30 min), providing (a) sufficient catching water molecules and hydrogen ions on the surface of the catalyst, and (b) stable transfer channel for electrons to repress the recombination of e^−^–h^+^ pairs [62]. In another approach, Xu et al. carried out a plasma treatment (N_2_/H_2_, atmosphere, 500 °C, 1400 W, and 30 min) for preparing layered *g*–C_3_N_4_/plasma-treated Ti_3_C_2_T_x_ photocatalyst [63]. Based on analyzed results by Raman, FTIR, and XPS, Xu et al. observed an increase of Ti–O with a decrease of Ti–C, Ti–F, and Ti–OH. Additionally, the plasma-treated Ti_3_C_2_T_x_ photocatalyst worked as an excellent acceptor of photogenerated electrons, leading to substantially reinforce the photocatalytic activity. Though the surface termination groups could be modified by several methods, such as hydrothermal treatment, simple calcination, plasma treatment, etc., more studies that elucidate the modification mechanism of surface termination groups need to be paid attention in the future.

## 4. The Design of Ti_3_C_2_T_x_ Composite Photocatalysts

### 4.1. Couple with Transition Metal Oxide (TMOs)

**Figure 5:**
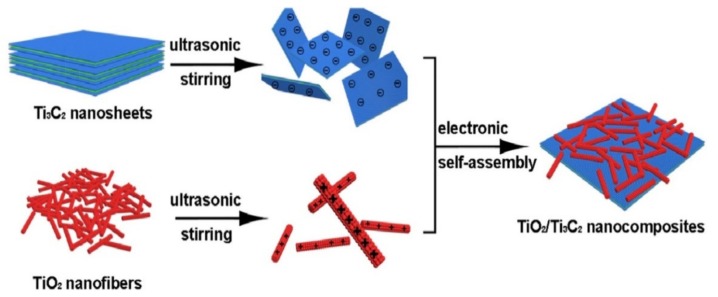
Schematic illustration displaying procedure for fabrication of TiO_2_/Ti_3_C_2_T_x_ composite. Reproduced with permission from reference [67]. Copyright Elsevier B.V., 2019.

Transition metal oxide, such as titanium dioxide (TiO_2_), coupled photocatalysts have attracted dramatically increasing interest in the area of photocatalytic hydrogen generation [64,65,66]. Their photocatalytic activities have been markedly improved through the efforts of many research groups. However, its large bandgap and fast charge recombination limit its efficiency. To overcome this limitation, Ti_3_C_2_T_x_ has been considered as promising cocatalysts for hydrogen production with TiO_2_ as the photocatalyst. Zhuang has successfully prepared TiO_2_/Ti_3_C_2_T_x_ nanocomposites by the electrostatic self-assembly technique (Figure 5) [67]. Owing to the highly efficient separation of photogenerated carriers, which derived from the intense interfacial contact between TiO_2_ nanofibers and Ti_3_C_2_T_x_ nanosheets, the photocatalytic performance over TiO_2_/Ti_3_C_2_T_x_ nanocomposites was significantly improved. The H_2_ production rate was up to 6979 μmol·g_cat_^−1^·h^−1^ using a 10% methanol solution as the sacrificial electron donors under a 300 W Xe lamp, which was 3.8 times higher than that of pure TiO_2_ nanofibers. There was no hydrogen production capacity over Ti_3_C_2_T_x_ nanosheets due to its metallic character.

To simplify the synthesis method, simple calcination was first proposed by Li et al. to prepare truncated octahedral bipyramidal TiO_2_ (TOB-T)/Ti_3_C_2_T_x_ hybrids [37]. The resultant TiO_2_/Ti_3_C_2_T_x_ hybrids retained the multilayer structure, and TiO_2_ exhibited a truncated octahedral bipyramidal structure with exposed (001) and (101) facets. A surface heterojunction between (101) and (001) facets was established, and it could prevent the recombination of photogenerated carriers in TiO_2_. Moreover, the remaining Ti_3_C_2_T_x_ could act as a cocatalyst to accelerate the migration of photoinduced electrons because of its high electronic conductivity. Meanwhile, the concentration of fluorine sharply decreased during calcination, thereby reducing the toxicity and increasing the conductivity of the samples. They pointed out that Ti_3_C_2_T_x_ could enhance the photocatalytic activity of those composite photocatalysts due to the Schottky junction between Ti_3_C_2_T_x_ and TiO_2_ and its excellent electronic conductivity. Besides TiO_2_, ZnO has also been investigated for hydrogen production [68]. It was experimentally demonstrated that the ZnO nanorods (NRs)/Ti_3_C_2_T_x_ hybrids exhibited the inferior photocatalytic H_2_ production activity (456 μmol·h^−1^), while pure ZnO NRs displayed no performance [68]. However, the photocatalytic activity of the ZnO/Ti_3_C_2_T_x_ composite was still much lower compared to the TiO_2_/Ti_3_C_2_T_x_, thus, more investigation is necessary.

### 4.2. Couple with Transient Metal Sulfides (TMSs)

**Figure 6:**
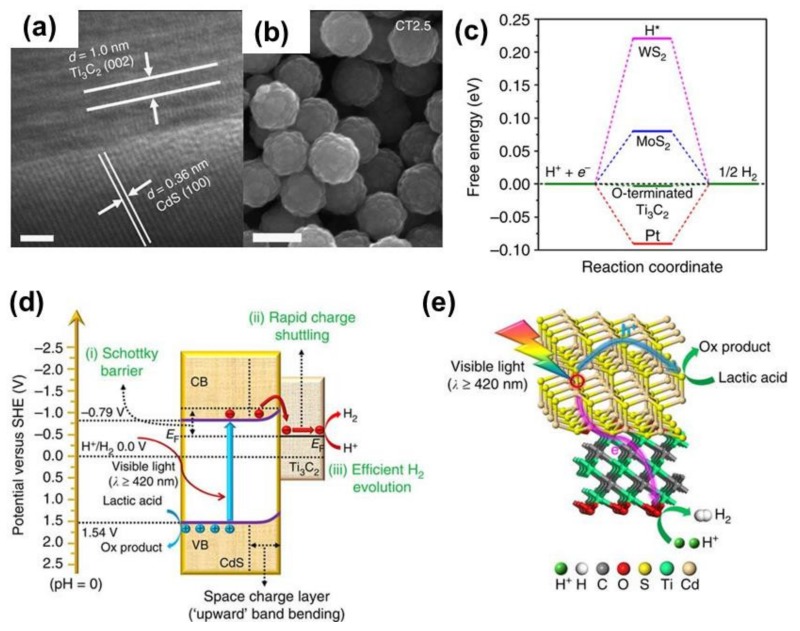
(**a**,**b**), TEM and SEM images of Ti_3_C_2_T_x_/CdS composite structure; (**c**) the calculated free-energy band diagram of HER with different catalysts including MoS_2_, WS_2_, and O-Ti_3_C_2_T_x_; (**d**) band diagram of Ti_3_C_2_T_x_/CdS showing the charge separation and transferring from CdS to Ti_3_C_2_T_x_ for HER; and (**e**) the proposed mechanism of HER over Ti_3_C_2_T_x_/CdS composite. Reproduced with permission from reference [61]. Copyright Nature Publishing Group, 2017.

Transition metal surface such as CdS [69,70,71], CdSe [72], MoS_2_ [73,74,75], and WS_2_ [76,77,78] has been demonstrated as potential catalysts for electrocatalytic and photocatalytic HER. Therefore, the coupling of these materials with Ti_3_C_2_T_x_ might produce the composite with unprecedented performance in photocatalytic HER. As expected, Ran et al. coupled O–Ti_3_C_2_T_x_ with cadmium sulfide (CdS) via a hydrothermal method to yield a composite catalyst for HER with very high performance [61]. In specific, the catalysts with the optimized composition (2.5 wt % Ti_3_C_2_T_x_) can produce up to 14,342 μmol·g_cat_^−1^·h^−1^, which was higher than that of Pt-CdS (10,978 μmol·g_cat_^−1^·h^−1^). The HR-TEM and SEM images of the O–Ti_3_C_2_T_x_ coupled CdS nanoparticles are shown in Figure 6a–b. The high photocatalytic HER performance of the O-Ti_3_C_2_T_x_/CdS composite attributed to very low free energy for atomic H adsorption on the surface of O–Ti_3_C_2_T_x_ (Figure 6c) and efficient charge generation and separation upon light at the interface of the composites (Figure 6d–e). Similarly, Xiao et al. coupled Ti_3_C_2_T_x_ with CdS nanorod to construct a Schottky heterojunction for photocatalytic HER [79]. As a result, the CdS nanorod/Ti_3_C_2_T_x_ nanosheet exhibited a performance 7-fold higher than that of pristine CdS [79]. The improvement was postulated to originate from the synergistic effect between the CdS nanorod and Ti_3_C_2_T_x_ nanosheets that improves light absorption, charge separation, and conductivity of the composite catalysts. Tie et al. decorated ZnS nanoparticles with Ti_3_C_2_T_x_ nanosheets to yield photocatalytic HER with a production rate of 502.6 μmol·g_cat_^−1^·h^−1^ under optimal conditions, is almost 4-fold higher than pure ZnS (124.6 μmol·g_cat_^−1^·h^−1^) [80]. Besides, the alloy transition metal sulfide/Ti_3_C_2_T_x_ was also investigated. For example, Cheng et al. demonstrated a high-performance composite for photocatalytic HER composed of CdLa_2_S_4_ and Ti_3_C_2_T_x_ nanocomposite [81]. In specific, these composite nanomaterials yield photocatalytic HER with the H_2_ production rate of 11,182.4 μmol·g_cat_^−1^·h^−1^, and apparent quantum efficiency reached 15.6% at 420 nm. The performance of CdLa_2_S_4_/Ti_3_C_2_T_x_ nanocomposite, therefore, improves the production rate up to 13.4 times compared to that of pristine CdLa_2_S_4_ and even higher than that of Pt/CdLa_2_S_4_. To sum up, Ti_3_C_2_T_x_ couple with TMSs could reach to a desirable level. In detail, 2.5 wt % Ti_3_C_2_T_x_/CdS and ZnS/Ti_3_C_2_T_x_ nanosheets exhibited very attractive photocatalytic activity, making them good candidates for photocatalytic HER.

### 4.3. Couple with the Metal–Organic Framework 

**Figure 7:**
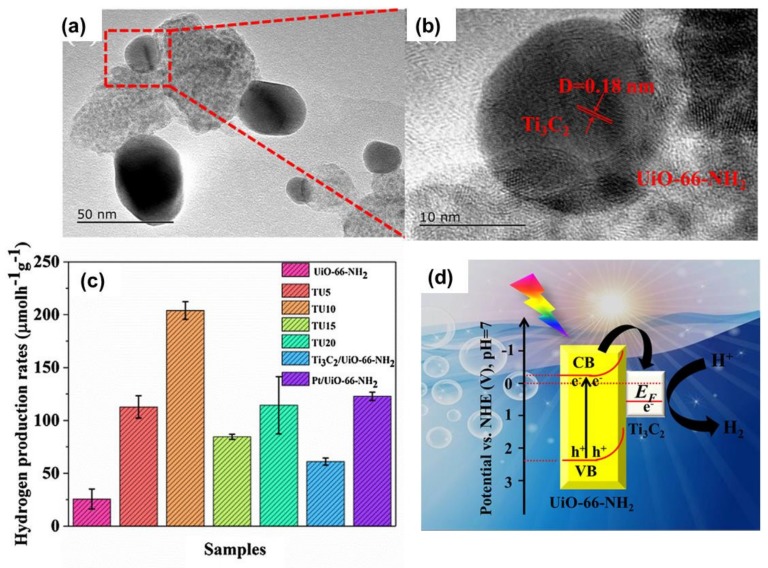
(**a**,**b**)TEM images presented the formation of Ti_3_C_2_T_x_ and Zr-MOFs heterostructure; (**c**) Hydrogen production rates of Ti_3_C_2_T_x_/Zr-MOF with different concentrations of Ti_3_C_2_T_x_; (**d**) Energy band diagram of Ti_3_C_2_T_x_/Zr-MOF for photocatalytic HER. Reproduced with permission from reference [82]. Copyright Elsevier B.V., 2019.

MOFs and their derivative have been emerging as efficient catalysts for photo electrocatalytic HER. The first combination of Ti_3_C_2_T_x_/MOFs composite was reported by Tian et al. in 2019 [82]. The TEM images in Figure 7a,b indicated that the MOFs were well connected with the MOFs. As a result, the Ti_3_C_2_T_x_/MOFs composite displays photocatalytic activity better than the Pt decorated MOFs (2 wt % Pt/UiO-66-NH_2_). The performance of Ti_3_C_2_T_x_/MOFs can be observed in Figure 7c. The schematic illustration of energy band alignment between Ti_3_C_2_T_x_ and MOFs is shown in Figure 7d. Under sunlight irradiation, the electron-hole pairs were generated in MOFs. Owing to the good contact and conductivity, the photo-induced electron can be easily transferred to the Ti_3_C_2_T_x_ surface to participate in the HER, thus, improving the overall performance of the composite catalysts.

### 4.4. Coupled with Graphitic Carbon Nitride (g–C_3_N_4_) 

**Figure 8:**
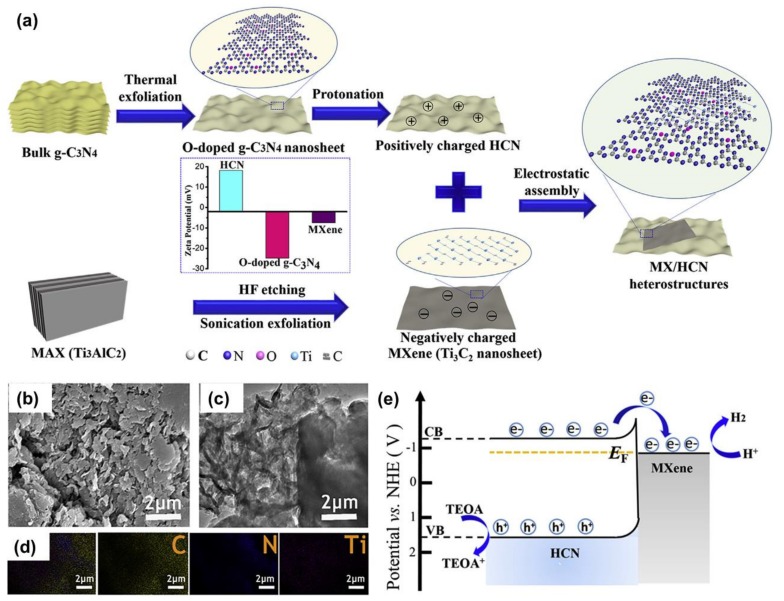
(**a**) Fabrication process of the Ti_3_C_2_T_x_/O-doped *g*–C_3_N_4_ heterostructure. (**b**–**d**) SEM images, TEM images, and EDS spectra of Ti_3_C_2_T_x_/O-doped *g*–C_3_N_4_. (**e**) The working mechanism of Ti_3_C_2_T_x_/O-doped *g*–C_3_N_4_ photocatalyst. Reproduced with permission from reference [87]. Copyright Elsevier B.V., 2019.

Graphitic carbon nitride (*g*–C_3_N_4_) coupled photocatalysts have attracted dramatically increasing interest in the area of visible-light-induced photocatalytic hydrogen generation due to the unique electronic band structure and high thermal and chemical stability of *g*–C_3_N_4_ [83,84,85]. Besides, the work had been done by Li et al. in the previous section, *g*–C_3_N_4_@Ti_3_C_2_T_x_ QDs [55], another study that couples Ti_3_C_2_T_x_/g–C_3_N_4_ has also been reported. Typically, Su et al. constructed a heterojunction using Ti_3_C_2_T_x_ and *g*–C_3_N_4_ nanosheets via the electrostatic self-assembly method [86]. A small amount of Ti_3_C_2_T_x_ was loaded onto *g*–C_3_N_4_, with a concentration that ranged from 1% to 5%. Interestingly, the Ti_3_C_2_T_x_/*g*–C_3_N_4_ exhibits significantly improved photocatalytic activity towards HER compared to that of pristine *g*–C_3_N_4_ [86]. Instead of using pristine *g*–C_3_N_4_, Lin et al. used O-doped *g*–C_3_N_4_ to form the heterostructure with Ti_3_C_2_T_x_ to improve the H_2_ production rate of catalysts two-fold [87]. The fabrication process for constructing Ti_3_C_2_T_x_/O-doped *g*–C_3_N_4_ is shown in Figure 8a. The SEM and TEM images in Figure 8b–d indicates that well interspersed Ti_3_C_2_T_x_/O-doped g–C_3_N_4_ heterostructure was obtained. As a result, the Ti_3_C_2_T_x_/O-doped *g*–C_3_N_4_ yield H_2_ with a production rate of 25,124 μmol·g_cat_^−1^·h^−1^, whereas, pristine O-doped *g*–C_3_N_4_ and Ti_3_C_2_T_x_/pristine *g*–C_3_N_4_ exhibit a lower H_2_ generation rate of 13,745 and 15,573 μmol·g_cat_^−1^·h^−1^, respectively. Figure 8e indicates that the electron from O-doped *g*–C_3_N_4_ can be easily transferred to Ti_3_C_2_T_x_ for the HER. These results suggested that *g*–C_3_N_4_ is a very good photoactive material to pair with Ti_3_C_2_T_x_ to yield efficient photocatalytic HER. However, the research related to this topic is still very limited, thus it needs more investigation in the near future. 

### 4.5. Ternary Composites

**Figure 9:**
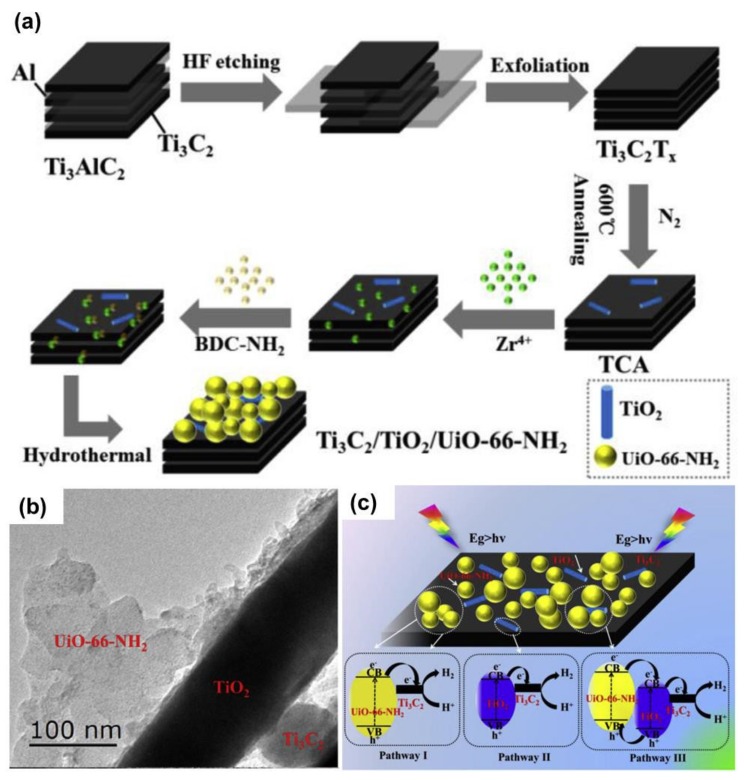
(**a**) Route for the synthesis of Ti_3_C_2_T_x_/TiO_2_/UiO-66-NH_2_ ternary composite, (**b**) TEM image of the Ti_3_C_2_T_x_/TiO_2_/UiO-66-NH_2_ ternary composite, and (**c**) working mechanism of ternary composite photocatalyst for HER. Reproduced with permission from reference [88]. Copyright Elsevier B.V., 2019.

Apart from binary composites, ternary composites of Ti_3_C_2_T_x_ have also been rationally developed. To obtain the ternary composite catalyst, Tial et al. first introduced TiO_2_ onto the surface of Ti_3_C_2_T_x_ via thermal annealing at 600 °C under N_2_ atmosphere [88]. After that, the Zr–MOF (UiO-66-NH_2_) was growth on Ti_3_C_2_T_x_/TiO_2_ using a facile hydrothermal approach. The schematic illustration of the synthesis procedure is shown in Figure 9a. The TEM displaying the ternary phase of the composite is presented in Figure 9b. It can be observed that the ternary structure was well established. As a consequence, the ternary composite (Ti_3_C_2_T_x_/TiO_2_/UIO-66-NH_2_) exhibited a performance two times higher than that of the binary composite (Ti_3_C_2_T_x_/UIO-66-NH_2_). The improvement in the catalytic activity of the Ti_3_C_2_T_x_/TiO_2_/UIO-66-NH_2_) not only comes from the improvement of the light absorption by using a double light absorber (TiO_2_/UIO-66-NH_2_) but also the enhancement of the charge separation of collection efficiency. The working mechanism of the binary and ternary composite was clearly illustrated in Figure 9c. Additionally, by taking advantage of the ternary composites with the composition of Mo_x_S/TiO_2_/Ti_3_C_2_T_x_, Li et al. improved the H_2_ production rate up to 10,505.8 μmol·g_cat_^−1^·h^−1^, which was 193 times compared to that of pristine TiO_2_ [46]. Similarly, many other ternary composites have been constructed with excellent photocatalytic activity towards HER such as Mo_x_S@TiO_2_@Ti_3_C_2_T_x_ [46], Cu/TiO_2_@Ti_3_C_2_T_x_ [89], 1T–MoS_2_/Ti_3_C_2_T_x_/TiO_2_ [90], 1T–WS_2_@TiO_2_@Ti_3_C_2_T_x_ [91], Cu_2_O/(001)/TiO_2_/Ti_3_C_2_T_x_ [92], Ti_3_C_2_T_x_/TiO_2_/*g*–C_3_N_4_ [93], *g*–C_3_N_4_/Ti_3_C_2_T_x_/Pt [45], CdS/MoS_2_/Ti_3_C_2_T_x_ [94], and TiO_2_/Ti_3_C_2_T_x_/CoS_x_ [95]. However, it is noted that a multicomponent photocatalytic hybrid composed of MXene with other cocatalysts are still in an early stage and requires further efforts.

## 5. Comparison of the Photocatalytic Hydrogen Production

To sum up, a detailed summary and comparison of recently reported Ti_3_C_2_T_x_ cocatalysts toward photocatalytic hydrogen production are given in Table 1. Although the experimental reaction conditions were different, we compared the photocatalytic activity in terms of the H_2_ evolution rate. Then, all the evolution rate of H_2_ were obtained and transformed into a logical unit (μmol·g_cat_^−1^·h^−1^) for acceptable comparative purposes. We found that Ti_3_C_2_T_x_/O-doped *g*–C_3_N_4_ achieved interest in the H_2_ evolution rate (25,124 μmol·g_cat_^−1^·h^−1^). To further understand the photocatalytic activity of MXenes, a broad comparison was collected for different types of MXenes (as shown in Table 2). In addition to Ti_3_C_2_T_x_, only a few studies using other types of MXenes cocatalysts, such as Nb_2_CT*_x_* [96] and Ti_2_C [97], for hydrogen production. Interestingly, the hybrid composite of Zn_0.5_Cd_0.5_S and Ti_2_C/TiO_2_ exhibited an attractive H_2_ production rate (32,560 μmol·g_cat_^−1^·h^−1^) [97]. This photocatalytic enhancement might be contributed by the effective light absorption and the efficient separation of electron-hole pairs.

## 6. Summary and Perspectives

In conclusion, Ti_3_C_2_T_x_ exhibited excellent catalytic properties toward photocatalytic HER. However, the property of Ti_3_C_2_T_x_ was strongly affected by its surface functional groups and coupled materials. Specifically, the O terminated Ti_3_C_2_T_x_ offered the best catalytic activity. The performance of Ti_3_C_3_T_x_ could also be improved by paring with other photoactive materials such as TiO_2_, ZnO, MoS_2_, WS_2_, CdS, and graphitic carbon nitride. The composite materials not only improved light absorption but also enhanced the charge separation and active sites. Thus improving the overall performance Ti_3_C_2_T_x_ under UV-vis light irradiation. Nonetheless, there were still limitations that hinder the application of Ti_3_C_2_T_x_ for practical applications such as scalability and stability. The future development of Ti_3_C_2_T_x_ as photocatalysts can be extended into the following directions: (1) developing a novel method for production of Ti_3_C_2_T_x_ in large scale at a mild condition such as a lower temperature, less toxic etchant, and solution-processable; (2) constructing novel functional groups on the surface of Ti_3_C_2_T_x_ for improving the catalytic properties; (3) designing novel materials to couple with Ti_3_C_2_T_x_ for further enhancing the photocatalytic activity such as oxide perovskite and halide perovskite can be considered; and (4) improving the stability of Ti_3_C_2_T_x_ for improving the lifetime of catalysts under working through structural engineering or passivation.

## Figures and Tables

**Table 1:** Photocatalytic hydrogen production over Ti_3_C_2_T_x_ cocatalysts.

No.	Photocatalysts	Light Source	Reaction Temp.	Scavenger	Reactant Medium	H_2_ Production Rate (μmol·g_cat_^−1^·h^−1^)	Ref/(Year)
1	TiO_2_ nanofibers/ Ti_3_C_2_T_x_ nanosheets (3 wt %)	300 W Xe lamp	Room temperature (RT)	Methanol	CH_3_OH/H_2_O (*l*, 1:9)	6979	[67]/2019
2	TiO_2_ nanofibers					1831	
3	Ti_3_C_2_T_x_ nanosheets					ND	
4	F–Ti_3_C_2_T_x_/TiO_2_ hybrids	350 W Xe arc lamp	RT	Glycerin	C_3_H_8_O_3_/H_2_O (*l*, 1:9)	127.1	[37]/2019
5	OH–Ti_3_C_2_T_x_/TiO_2_ hybrids					61.4	
6	CdS (CT0)	300 W Xe arc lamp: λ ≥ 420 nm; 80 mW·cm^−2^	RT	Lactic acid	C_3_H_6_O_3_/H_2_O (*l*, 17.6:62.4)	105	[61]/2017
7	Ti_3_C_2_T_x_ nanoparticles					ND	
8	0.05 wt % Ti_3_C_2_T_x_ nanoparticles/CdS (CT0.05)					993	
9	0.1 wt % Ti_3_C_2_T_x_ nanoparticles/CdS (CT0.1)					1278	
10	2.5 wt % Ti_3_C_2_T_x_ nanoparticles/CdS (CT2.5)					14,342	
11	5 wt %Ti_3_C_2_T_x_ nanoparticles/CdS (CT5)					3377	
12	Pt/CdS					10,978	
13	NiS/CdS					12,953	
14	Ni/CdS					8649	
15	MoS_2_/CdS					6183	
16	Ti_3_C_2_T_x_ nanosheets modified Zr–MOFs (UiO-66-NH_2_)	350 W Xe lamp	RT	S^2−^/SO_3_^2−^	0.1 M Na_2_S and 0.1 M Na_2_SO_3_	204	[82]/2019
17	2 wt % Pt/UiO-66-NH_2_					123	
18	UiO-66-NH_2_					25.6	
19	Zn_2_In_2_S_5_/Ti_3_C_2_T_x_ hybrids	300 W Xe arc lamp: λ ≥ 420 nm;	RT	S^2−^/SO_3_^2−^	0.35 M Na_2_S and 0.25 M Na_2_SO_3_	2596.8	[92]/2019
20	Ti_3_C_2_T_x_/TiO_2_/UiO-66-NH_2_ hybrid	300 W Xe lamp (PerkinElmer): 350 < λ < 780 nm	5 °C	S^2−^/SO_3_^2−^	0.1 M Na_2_S and 0.1 M Na_2_SO_3_	1980	[88]/2019
21	Ti_3_C_2_T_x_/UiO-66-NH_2_					1320	
22	UiO-66-NH_2_					942.9	
23	MoxS@TiO_2_@Ti_3_C_2_T_x_ composite	300 W Xe arc lamp: an AM1.5 filter; 180 mW·cm^−2^ within a range of 200–1200 nm.	25 °C	Triethanolamine (TEOA)	TEOA in aqueous acetone	10505.8	[46]/2020
24	Cu/TiO_2_@Ti_3_C_2_T_x_	300W Xe lamp (CEL-HXF 300E)	RT	Methanol	CH_3_OH/H_2_O (*l*, 1:14)	764	[89]/ 2018
25	TiO_2_@Ti_3_C_2_T_x_					65	
26	1T–MoS_2_ nanopatch/Ti_3_C_2_T_x_/TiO_2_ nanosheet	300 W Xe arc lamp: an AM1.5 filter; 180 mW·cm^−2^ within a range of 200–1200 nm.	25 °C	TEOA	TEOA/Acetone/H_2_O (*l*, 1:3:16)	9738	[90]/2019
27	Ti_3_C_2_T_x_/TiO_2_ nanosheet					898	
28	TiO_2_ nanosheet					74	
29	1T–WS_2_@TiO_2_@ Ti_3_C_2_T_x_	300 W Xe arc lamp: an AM-1.5 filter	25 °C	TEOA	TEOA/Acetone/H_2_O (*l*, 1:3:16)	3409.8	[91]/2019
30	TiO_2_					67.8	
31	ternary Cu_2_O/(001) TiO_2_@Ti_3_C_2_T_x_	300 W Xe lamp (CEL-HXF 300E)	RT	Methanol	CH_3_OH/H_2_O (*l*, 1:14)	1496	[92]/2019
32	(001) TiO_2_@ Ti_3_C_2_T_x_					165	
33	Ti_3_C_2_T_x_@TiO_2_@MoS_2_ composites	300 W Xe arc lamp (CELHXF300): an AM1.5 filter	25 °C	TEOA	TEOA in aqueous acetone	6425.3	[95]/2019
34	Ti_3_C_2_T_x_@TiO_2_					898.1	
35	TiO_2_/Ti_3_C_2_T_x_/CoS	300 W Xe arc lamp	RT	Methanol	CH_3_OH/H_2_O (*l*, 1:4)	950	[95]/2019
36	TiO_2_					140	
37	CoS					10	
38	TiO_2_/Ti_3_C_2_T_x_					330	
39	TiO_2_/CoS					540	
40	*g*–C_3_N_4_/Ti_3_C_2_T_x_/Pt	300 W Xe arc lamp	RT	TEOA	TEOA/H_2_O (*l*, 1:9)	5100	[45]/2018
41	*g*–C_3_N_4_/Ti_3_C_2_T_x_					1700	
42	*g*–C_3_N_4_/Pt					1275	
43	*g*–C_3_N_4_@Ti_3_C_2_T_x_ quantum dots	300 W Xe arc lamp (CELHXF300): an AM-1.5 filter	RT	TEOA	TEOA/H_2_O (*l*, 3:17)	5111.8	[55]/2019
44	*g*–C_3_N_4_					196.8	
45	Pt/*g*–C_3_N_4_					1896.4	
46	Ti_3_C_2_T_x_/O-doped *g*–C_3_N_4_	300 W Xe lamp	RT	TEOA	TEOA (*l*)	25,124	[87]/2019
47	O-doped *g*–C_3_N_4_					13,745	
48	Ti_3_C_2_T_x_/*g*–C_3_N_4_					15,573	
49	Ti_3_C_2_T_x_/TiO_2_/*g*–C_3_N_4_ nanocomposites	300 W Xe lamp: λ > 420 nm	25 °C	TEOA	TEOA/H_2_O (*l*, 2:17)	1620	[93]/2018
50	*g*–C_3_N_4_					670	
51	CdLa_2_S_4_/Ti_3_C_2_T_x_ nanocomposite	300 W Xe lamp: a high-pass filter (λ > 420 nm)	RT	S^2−^/SO_3_^2−^	0.35 M Na_2_S and 0.25 M Na_2_SO_3_	11,182.4	[81]/2019
52	Pt/CdLa_2_S_4_					1734.7	
53	CdLa_2_S_4_					832	
54	Ti_3_C_2_T_x_					ND	
55	CdS nanorod/ Ti_3_C_2_T_x_ nanosheet	300 W Xe lamp (PerkinElmer): a cut-off filter (λ > 420 nm)	6 °C	Lactic acid	C_3_H_6_O_3_/H_2_O (*l*, 1:9)	2407	[79]/2019
56	CdS nanorod					360	
57	ZnS nanoparticles/Ti_3_C_2_T_x_ nanosheets	300 W Xe lamp	RT	Lactic acid	C_3_H_6_O_3_/H_2_O (*l*, 1:4)	502.6	[80]/2019
58	ZnS nanoparticles					124.6	
59	ZnO nanorods /Ti_3_C_2_T_x_ hybrids	300 W Xe lamp: λ > 420 nm	RT	Ethanol	C_2_H_5_OH/H_2_O (*l*, 3:16)	456	[68]/2020
60	ZnO nanorods					ND	
61	CdS/MoS_2_/Ti_3_C_2_T_x_ composites	300 W Xe lamp (CELHXF300): a cut-off filter (λ > 420 nm)	RT	S^2−^/SO_3_^2−^	0.25 M Na_2_S and 0.35 M Na_2_SO_3_	9679	[94]/2019
62	plasma-Ti_3_C_2_T_x_/CdS hybrids	300 W arc Xe lamp (PLSSXE300): a UV cut-off filter (λ > 420 nm);	RT	Lactic acid	C_3_H_6_O_3_/H_2_O (*l*, 1:9)	825	[62]/2019
63	Ti_3_C_2_T_x_/CdS hybrids					473	
64	*g*–C_3_N_4_/plasma-Ti_3_C_2_T_x_	350 W Xe lamp: a UV cut-off filter (λ > 400 nm); 70 mW·cm^−2^	RT	TEOA	TEOA/H_2_O (*l*, 1:9)	17.8	[63]/2020
65	*g*–C_3_N_4_/Ti_3_C_2_T_x_					7.5	
66	*g*–C_3_N_4_					0.7	
67	TiO_2_/Ti_3_C_2_T_x_@AC-48 h composite	350 W Xe lamp (AHD 350): a cut-off filter (λ > 400 nm)	RT	Ascorbic acid (AA)	29 mg·mL^−1^ AA with the sensitization of 1 mM EY in aqueous solution	33.4	[98]/2019
68	1% Pt/TiO_2_					0.7	
69	TiO_2_/Ti_3_C_2_T_x_@AC-48 h composite				29 mg·mL^−1^ AA in aqueous solution	0.3	

**Table 2:** Photocatalytic hydrogen production over selected MXenes cocatalysts.

No.	Photocatalysts	Light Source	Reaction Temp.	Scavenger	Reactant Medium	H_2_ Production Rate (μmol·g_cat_^−1^·h^−1^)	Ref./(Year)
1	Ti_3_C_2_T_x_/O-doped *g*–C_3_N_4_	300 W Xe lamp	RT	TEOA	TEOA (*l*)	25,124	[87]/2019
2	CdLa_2_S_4_/Ti_3_C_2_T_x_ nanocomposite	300 W Xe lamp: a high-pass filter (λ > 420 nm)	RT	S^2−^/SO_3_^2−^	0.35 M Na_2_S and 0.25 M Na_2_SO_3_	11,182.4	[81]/2019
3	2.5 wt % Ti_3_C_2_T_x_ nanoparticles/CdS (CT2.5)	300 W Xe arc lamp: λ ≥ 420 nm; 80 mW·cm^−2^	RT	Lactic acid	C_3_H_6_O_3_/H_2_O (*l*, 17.6:62.4)	14,342	[61]/2017
4	Nb_2_O_5_/C/Nb_2_CT*_x_* Composites	200 W Hg lamp: λ = 285–325 nm; 120 mW·cm^−2^	25 °C	Methanol	CH_3_OH/H_2_O (*l*, 1:3)	7.81	[96]/2018
5	Zn_0.5_Cd_0.5_S/Ti_2_C/TiO_2_	300 W Xe lamp: λ ≥ 400 nm;	RT	S^2−^/SO_3_^2−^	0.3 M Na_2_S and 0.3 M Na_2_SO_3_	32,560	[97]/2020
